# Drug-Metabolizing Enzymes and Metabolic Diseases: Role of Antioxidants

**DOI:** 10.1155/2016/2026582

**Published:** 2016-11-13

**Authors:** Salah A. Sheweita, Mahmoud Balbaa, Samy L. Habib

**Affiliations:** ^1^Alexandria University, Alexandria, Egypt; ^2^University of Texas Health Science Center, San Antonio, TX, USA

Drug-metabolizing enzymes include many cytochrome P450 isozymes and others. The hepatic cytochromes P450 (CYP) are a multigene family of enzymes that metabolize many drugs and carcinogens. Recently, natural and synthetic antioxidants were found to reduce the incidence of tumors since the decrease in antioxidant levels is correlated with the severity of the malignancy of tumors. In addition, the formations of considerable amounts of oxidative stress products including free radicals are responsible for induction of many diseases including cancers, diabetes, and infertility. In this special issue on drug-metabolizing enzymes and metabolic diseases, six papers address such issue.

In this special issue, S. A. Sheweita et al. addressed the effects of erectile dysfunction (ED) on the activity of drug-metabolizing enzymes including cytochrome P450s (CYPs). They have shown that low doses of tadalafil and vardenafil induced the protein expression of CYP2E1 whereas high doses of either tadalafil or sildenafil were more potent inhibitors to CYP2E1 expression than vardenafil. In addition, such drugs inhibited the protein expression of CYP B1/2 along with its corresponding enzyme marker activity. They have found that the changes in the expression and activity of phase I drug-metabolizing enzymes could change the normal metabolic pathways and might enhance the deleterious effects of exogenous as well as endogenous compounds. R. B. Freitas et al. showed the effects of* Euterpe edulis* bioproducts (lyophilized pulp, defatted lyophilized pulp, and oil (pulp oil)) on nonalcoholic fatty liver disease induced by a high-fat diet in rats. They found that dietary intake of lyophilized pulp and especially defatted lyophilized pulp, but not EO, attenuated diet-induced nonalcoholic fatty liver disease, reducing inflammatory infiltrate, steatosis, and lipid peroxidation in liver tissue. M. Balbaa et al. have found that* Nigella sativa* oil significantly induced the gene expression of insulin receptor and upregulated the expression of insulin-like growth factor-1 and phosphoinositide-3 kinase. In addition, the* Nigella sativa* oil reduced blood glucose level, components of the lipid profile, oxidative stress parameters, serum insulin/insulin receptor ratio, and the tumor necrosis factor-*α*, in rats, and indicated that* Nigella sativa* oil had a potential in the management of diabetes as well as improvement of insulin-induced signaling.

J. Zhang et al. showed that the relative reactive oxygen species (ROS) level and DNA oxidative damage were significantly increased in the cell line with inhibited glucose 6-phosphate dehydrogenase (G6PD) upon treatment with 1,4-benzoquinone (BQ). The apoptotic rate and G2 phase arrest were also significantly higher in the cells with inhibited G6PD and exposed to BQ than in the control cells. G6PD inhibition could reduce GSH activity and alleviate oxidative damage. G6PD deficiency was also a possible susceptible risk factor of benzene exposure. S. Ficarra et al. addressed the short-term effects of chlorpromazine on erythrocytes. They have found that chlorpromazine destabilized and increased the autooxidation of hemoglobin, induced activation of caspase 3, increased phosphatase PTP-1B activity, and, markedly, influenced the ATP and reduced glutathione levels, with subsequent exposure of phosphatidylserine at the erythrocyte surface. Overall, the early stage of chlorpromazine influence on erythrocytes could contribute to better understanding of new and interesting characteristics of this compound. I. Pérez-Torres et al. showed that estradiol deficiency increased retroperitoneal fat and large adipocytes. However, cross-sex hormonal replacement in female rats aggravated the condition by inhibiting antioxidant enzymes.

*Salah A. Sheweita* *Salah A. Sheweita* *Mahmoud Balbaa* *Mahmoud Balbaa* *Samy L. Habib* *Samy L. Habib*

